# Recognizing Minor Leukemic Populations with Monocytic Features in Mixed-Phenotype Acute Leukemia by Flow Cell Sorting Followed by Cytogenetic and Molecular Studies: Report of Five Exemplary Cases

**DOI:** 10.3390/ijms24065260

**Published:** 2023-03-09

**Authors:** Alexandra Semchenkova, Elena Zerkalenkova, Irina Demina, Svetlana Kashpor, Egor Volchkov, Elena Zakharova, Sergey Larin, Yulia Olshanskaya, Galina Novichkova, Alexey Maschan, Michael Maschan, Alexander Popov

**Affiliations:** 1 Dmitry Rogachev National Medical Research Center of Pediatric Hematology, Oncology and Immunology, 117198 Moscow, Russia; 2 Research Institute of Molecular and Cellular Medicine, Peoples’ Friendship University of Russia (RUDN University), 117198 Moscow, Russia

**Keywords:** flow cell sorting, mixed-phenotype acute leukemia, fluorescence in situ hybridization

## Abstract

Mixed-phenotype acute leukemia (MPAL), a rare and heterogeneous category of acute leukemia, is characterized by cross-lineage antigen expression. Leukemic blasts in MPAL can be represented either by one population with multiple markers of different lineages or by several single-lineage populations. In some cases, a major blast population may coexist with a smaller population that has minor immunophenotypic abnormalities and may be missed even by an experienced pathologist. To avoid misdiagnosis, we suggest sorting doubtful populations and leukemic blasts and searching for similar genetic aberrations. Using this approach, we examined questionable monocytic populations in five patients with dominant leukemic populations of B-lymphoblastic origin. Cell populations were isolated either for fluorescence in situ hybridization or for clonality assessment by multiplex PCR or next-generation sequencing. In all cases, monocytic cells shared the same gene rearrangements with dominant leukemic populations, unequivocally confirming the same leukemic origin. This approach is able to identify implicit cases of MPAL and therefore leads to the necessary clinical management for patients.

## 1. Introduction

Mixed-phenotype acute leukemia (MPAL) is a rare category of hematologic malignancy that accounts for up to 5% of all newly diagnosed acute leukemia cases [1]. These malignancies show no clear evidence of belonging to a single hematopoietic lineage and have worse outcomes compared to pure acute lymphoblastic (ALL) or myeloid (AML) leukemia [2,3,4,5]. Along with undifferentiated leukemia, which shows no lineage-specific antigens, MPAL was grouped into acute leukemia of ambiguous lineage (ALAL) in the World Health Organization (WHO) classification system in 2008 [6]. That edition summarized the diagnostic criteria for MPAL and defined specific genetic subgroups (*KMT2A*-rearranged and *BCR::ABL1*-positive MPALs) [6]. After some modifications in the 2016 [7] and 2022 [8] updates, this approach is now widely used in clinical practice.

Diagnosis of MPAL is mainly based on flow cytometry data, with the contribution of immunohistochemistry and cytochemistry in some cases [9]. Leukemic blasts can comprise a single population with a mixed antigen expression profile (biphenotypic leukemia) or represent two more or less distinct populations of blasts (bilineal leukemia) [10]. Although separated in relatively old classifications [11], these entities are now joined together in current WHO algorithms [6]. Correct interpretation of lineage markers on a single population of blasts is described thoroughly in the WHO classification [6]. When two separate populations are found in one specimen, each population should independently fulfill immunophenotypic criteria for either AML or B-/T-cell ALL [7]. In general, bilineal leukemias can be very problematic even for experienced pathologists [9,10,12,13,14], and one of the populations may exhibit an immunophenotype close to normal. For example, abnormal monoblasts sometimes resemble normal monocytes and cannot be distinguished from them only by immunophenotype because of the absence of specific immunophenotypic deviations [9,15]. In such a case, a small abnormal population may not be included in the final report, leading to misdiagnosis or missed diagnosis. A more detailed genetic study of doubtful populations and their comparison to unambiguous leukemic blasts of the same patient may help to verify bilineal leukemia. This can be accomplished by flow sorting followed by suitable molecular assays. Sorting of populations in MPAL has typically been applied mainly in research settings, as it is useful for detailed genetic characterization of leukemic cells [16,17,18]. However, we emphasize its importance in diagnostic routine for cases when the presence of MPAL is uncertain, but suspicious cell populations (even very small) are present in addition to the bulk leukemia. Herein, we report five cases of nonobvious bilineal MPAL presentation in patients with dominant B-lymphoblastic leukemia clarified through molecular investigations of sorted cell populations.

## 2. Results

**Figure 1:**
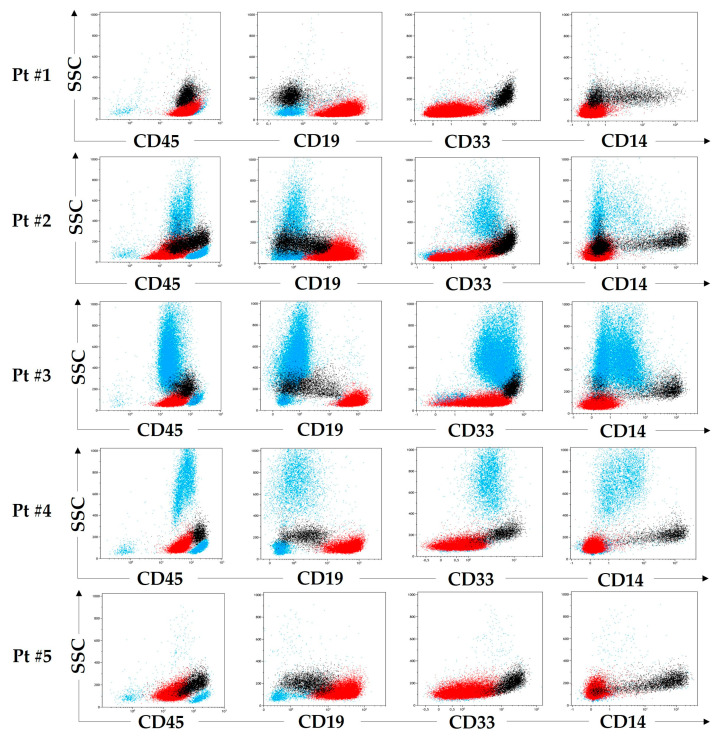
Review of key immunophenotypic features of leukemic B-lymphoblasts (red) and questionable monocyte-like cells (black) in five diagnostic samples. Other nucleated cells are shown in blue.

For all five patients, a small population of monocyte-like cells (Figure 1, black dots) accompanied the major leukemic population of B-lineage lymphoblasts. These cells were defined as suspicious because of partial CD19 positivity, which was significantly lower than that of B-lymphoblasts (Figure 1, red dots). The cells were sorted (B-lymphoblasts sorted in parallel as the control cells) both for fluorescence in situ hybridization (FISH) and for clonality studies because no recurrent genetic abnormalities were known at the time of sorting. As the most specific method, FISH was employed as the first-line method of confirmation; other molecular approaches were applied if no specific abnormalities were found in the whole bone marrow (BM). The results are summarized in Table S1.

### 2.1. Patient #1

**Figure 2:**
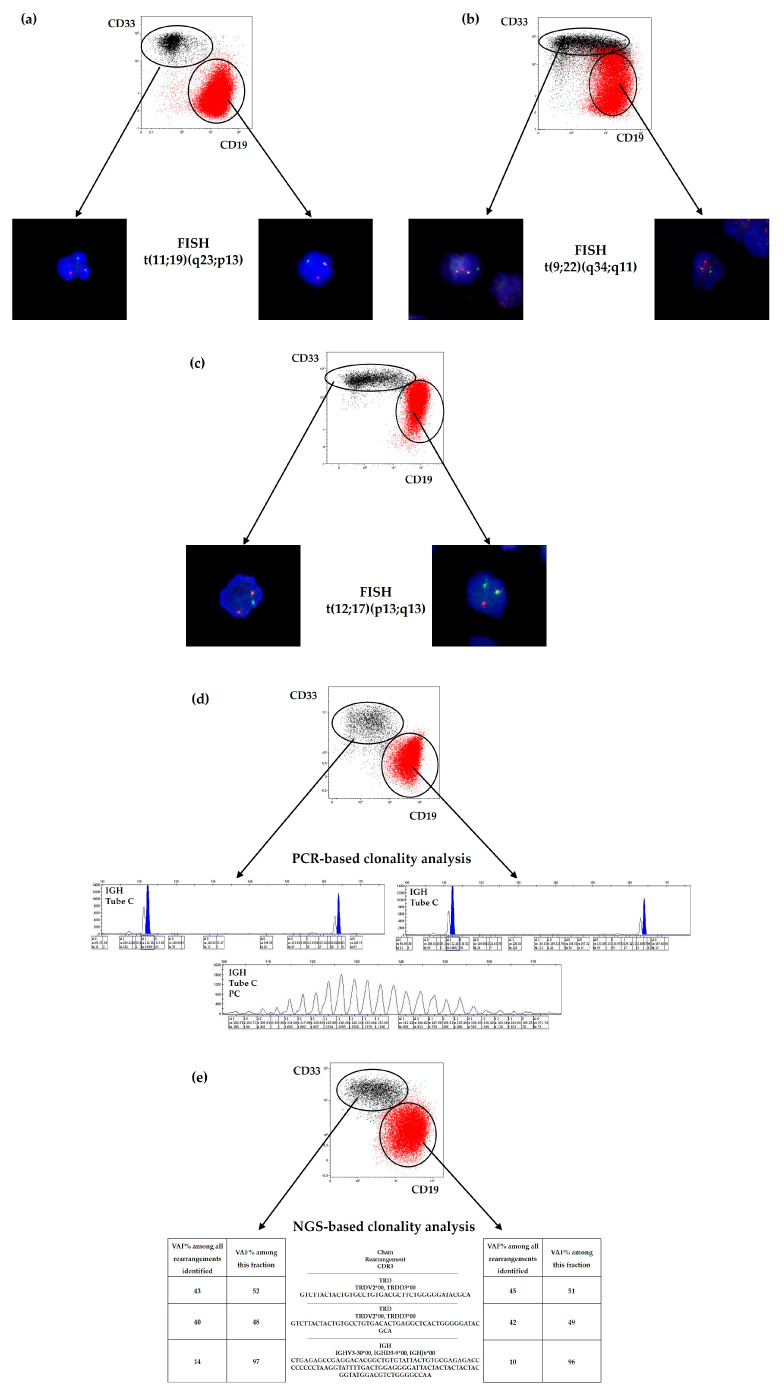
Confirmation of nonobvious bilineal cases of mixed-phenotype acute leukemia by cell sorting and molecular studies. Leukemic B-lymphoblasts (red) and monocyte-like cells (black) were isolated from diagnostic samples to confirm their common leukemic origin. In cases (**a**–**c**), FISH analysis of both cell populations confirmed the presence of specific rearrangements found previously in the bone marrow. In two other cases, the cells harbored identical *IG/TR* rearrangements demonstrated by either multiplex PCR (**d**) or NGS (**e**).

A 5-month-old female infant had only one population of blast cells according to the morphological study of BM smears. The blasts had clear lymphoid features and were negative for cytochemical staining. Immunophenotypically, 90% of all BM cells were positive for CD45, CD19, CD79a, CD15 (partial), and NG2 (partial) and negative for CD10 and CD22. We also identified a small population of cells (7%) positive for monocytic/myeloid markers (CD33, CD14, CD64, lysozyme, CD15) and with higher SSC. BM cytogenetics revealed one clone with a *KMT2A* rearrangement t(11;19)(q23;p13) confirmed by FISH. Therefore, both populations were isolated for FISH analysis, and *KMT2A* rearrangement was found in all sorted cells (Figure 2a).

### 2.2. Patient #2

An 11-year-old boy presented with two morphologically different blast populations on BM smears. The majority of blasts (60%) were medium-to-large in size, had variable nucleus-to-cytoplasm (N:C) ratios and were positive for MPO, SBB and ANAE. Approximately 25% of all blasts were small, had round nuclei and had a high N:C ratio. This portion of blasts was also negative for all of the cytochemical stains used. Flow cytometry revealed two large overlapping populations. One (42%) consisted of cells positive for CD45, CD19, CD10, CD34, CD79a, and CD13. Another population (38%) expressed various myeloid antigens (CD33, CD14, CD15, MPO) but was also partially positive for CD34, CD117, CD19, and CD79a. Cytogenetic analysis failed due to poor BM quality and lack of metaphases. However, FISH analysis confirmed the presence of t(9;22)(q34;q11)/*BCR::ABL1*. This translocation was also found in both sorted populations (Figure 2b).

### 2.3. Patient #3

BM smears also revealed lymphoid and myeloid blasts in the sample from another 3-month-old girl. Lymphoid blasts were predominant (67–82%): they were small, had a high N:C ratio and were positive only for PAS. A smaller population (3–10%) consisted of large blasts with round nuclei and a medium N:C ratio and tested positive for MPO, SBB, and PAS. Immunophenotyping demonstrated that 42% of all nucleated cells were positive for CD45, CD19, CD22, CD34, CD13 and CD33 but negative for CD10. In addition, 7% of the BM cells were represented by a monocytic/myeloid population positive for CD45, CD33, CD14, CD64, CD15, MPO and lysozyme. This population also expressed low levels of CD19 and CD22. According to cytogenetics, patient #3 had one clone harboring t(12;17)(p13;q11) with a *ZNF384* rearrangement, which was later found in sorted B-lymphoblastic and myeloid populations (Figure 2c).

### 2.4. Patient #4

BM aspirates of patient #4, an 18-year-old male, also showed one population of anaplastic blasts negative for MPO, SBB, ANAE, and PAS. According to flow cytometry, 65% of the BM cells consisted of lymphoblasts positive for CD45, CD19, CD10 (dim), and iCD79a and negative for CD22 and myeloid markers. A small population of monocyte-like cells (7%, positive for CD33, CD14, CD64, CD13 and lysozyme) displayed an atypically wide distribution on the CD19 vs. CD45 plot and somewhat overlapped with leukemic cells on some plots. Karyotyping did not reveal any BM abnormalities (46, XY). Blasts and monocyte-like cells were sorted for analysis of *IG/TR* rearrangements by multiplex PCR, and clonal *IGH* rearrangements were found in both cases (Figure 2d).

### 2.5. Patient #5

A 15-year-old female presented with BM smears completely infiltrated with blast cells of two different types. Small blasts with round nuclei, basophilic cytoplasm and a high N:C ratio constituted 89% of all blasts observed. The cells were negative for most cytochemical stains. Additionally, there were 11% intermediate-sized blasts with variable nuclei and N:C ratios positive only for SBB. Immunophenotyping also showed a population of lymphoblasts comprising 85% of total cells. The blasts were positive for CD45, CD19, CD10 (dim), CD22, CD79a and negative for any myeloid markers. A population of what was first considered to be monocytes (12%) expressed CD45 (bright), CD33, CD14, CD13 and lysozyme. However, the cells also partially expressed CD19. Cytogenetic analysis of the BM cells revealed a normal karyotype (46, XX). Considering the absence of any specific genetic abnormalities, two populations (leukemic B-lymphoblasts and monocyte-like cells) were sorted for analysis of clonal *IG/TR* rearrangements by high-throughput NGS. Both populations showed identical rearrangements of *TRD* and *IGH* loci (Figure 2e).

## 3. Discussion

In our study, we demonstrate the applicability of flow cell sorting with the subsequent use of various molecular techniques for confirmation of the leukemic origin of mature monocytes found in diagnostic BM samples together with the main leukemic B-lymphoblastic population. The results of these additional investigations allowed us to diagnose MPAL. In all cases, only slight positivity for CD19 (lower CD19 expression than that found in the B-lineage population) focused attention on these cells. All other antigens regularly used for BM immunophenotyping were expressed similarly to the elements of normal monocytic maturation [19,20,21]. In our study, the most common molecular aberrations associated with MPAL (*BCR::ABL1*, *KMT2A*-r and *ZNF384*-r) [8] were able to be studied by FISH in isolated monocytic populations with equal success as in major lymphoblastic populations. As the mentioned genetic rearrangements cover the vast majority of B/myeloid MPALs [18], the suggested method of MPAL confirmation (flow cell sorting plus FISH) may be extremely useful. In patients without specific genetic lesions, the leukemic origin of suspicious monocytes can be confirmed by assessing *IG/TR* gene rearrangements either with a sophisticated NGS-based approach or with simple RQ-PCR. The latter is possible because at the initial diagnostic stage, it is informative to simply detect the same types of *IG/TR* gene rearrangements in monocytic cells as in B-lymphoblasts.

For ages, it was considered that among all ALAL subtypes, only biphenotypic leukemia represents a diagnostic challenge but that the presence of two well-separated leukemic subpopulations is clearly visible during routine flow cytometric BM investigation [14,22]. Although biphenotypic and bilineal ALs were combined into a single MPAL category in the 2008 revision by the WHO [6], the established MPAL definition mainly covered cases with a single tumor population with immunophenotypic signs of different lineages. At the same time, in more or less typical cases of bilineal leukemia, each population can often be assigned to only one lineage and does not fulfill the relatively strict MPAL criteria [23]. Therefore, in the next revision of the WHO classification, a corresponding statement was added [7], allowing for the diagnosis of MPAL in bilineal leukemia cases. It is now clearly known that B/myeloid AL (with or without specific genetic aberrations) is the most common type of MPAL [4,18]. In most of these cases, an immature lymphoblastic population is accompanied by a more mature, predominantly monocytic population [9,15]. However, these monocytes typically do not show strong immunophenotypic aberrations. They mainly fit the known patterns of monocytic maturation [19], with a significant proportion of the cells displaying the antigen profile of monocytes rather than that of monoblasts. If the number of such cells is significantly lower than the number of B-lymphoblasts, it is always difficult to confirm the diagnosis of MPAL.

The precise diagnosis of MPAL has important clinical application. There are currently no protocols specifically designed for the treatment of MPAL, and the therapeutic regimen used is chosen depending on dominant lineage [4]. However, MPAL therapy often combines elements of ALL-directed and AML-directed therapy, whereas if malignant monocytic-like cells are not considered to be part of leukemia, only ALL therapy will be used. Moreover, algorithms for MRD monitoring are different for MPAL and pure ALL. Finally, the use of modern therapeutic approaches (e.g., immunotherapy) needs to be adjusted if there are two parts of leukemia. For example, it is now known that the use of blinatumomab or CAR-T cells can cause lineage switch, in particular, through selection of pre-existing myeloid leukemic subpopulation [24]. This possibility directly affects the choice of chemotherapy elements used together with immunotherapy [25]. Therefore, a clear distinction between MPAL and ALL cases is clinically important, and the combination of flow cytometry, flow cell sorting and molecular studies can provide valuable data.

Flow cell sorting is frequently used in MPAL investigations for scientific purposes [17,18]. For example, M. Kotrova et al. demonstrated the potential of high-speed cell sorting to confirm the genetic relationship of subpopulations in bilineal leukemia [17]. Nevertheless, attempts to implement this technique in routine practice of diagnostic laboratories are very rare [26,27]. Previously, we demonstrated the capabilities of cell sorting in assessing minimal residual disease in patients with acute leukemia after allogeneic HSCT or targeted therapy [28,29]. Its combination with different molecular techniques renders it a powerful instrument to be used in cases of difficult diagnosis. Although the limited number of cases is described in the current work, we suggest that a relatively easy and inexpensive procedure of flow cell sorting supplemented by FISH or clonality testing can be useful as an additional technique in routine clinical practice for the diagnosis of B/myeloid MPAL in the presence of low numbers of suspicious cells with a monocytic immunophenotype. Low partial (even on a minority of cells) expression of CD19 may serve as the main indicator of a need for such additional studies of BM monocytes. Sorting for both FISH and PCR allows for flexible choice of a preferable method of further investigation, depending on the results of cytogenetic and molecular diagnostics.

## 4. Materials and Methods

### 4.1. Patients and Samples

We describe five newly diagnosed patients with suspected ALL whose BM aspirates showed a population of leukemic B-lymphoblasts and a small population of suspicious monocyte-like cells. The patients were ≤18 years of age (median age 11 years, range 3 months—18 years); two of them were infants (<1 year of age).

### 4.2. Morphology and Cytochemistry

BM aspirate smears were stained with Wright–Giemsa for morphological analysis. The French–American–British (FAB) classification was used for the characterization of blasts [30]. Cytochemical stains used included myeloperoxidase (MPO), Sudan Black B (SBB), nonspecific esterase with alpha-naphthyl acetate as substrate (ANAE), and periodic acid-Schiff (PAS).

### 4.3. Flow Cytometry

Immunophenotyping was performed with antibody panels recommended by the Moscow–Berlin group [31]. Data were collected using Navios (Beckman Coulter, BC, Indianapolis, IN, USA) and FACS Canto II (Becton Dickinson, BD, San Jose, CA, USA) flow cytometers. EuroFlow guidelines for machine performance monitoring were applied [32]. Flow-Check Pro Fluorospheres (BC) and Cytometer Setup and Tracking Beads (BD) were used for daily instrument quality control. Data obtained were analyzed using Kaluza Analysis 2.1 software (BC). At least 20,000 cells were collected in the blast region defined according to CD45 expression and side-scatter (SSC) values. Membrane antigen positivity was defined if at least 20% of cells showed positive binding; cytoplasmic antigen positivity was defined as 10% positive cells.

### 4.4. Flow Cell Sorting

Diagnostic samples were processed for cell sorting. Suitable combinations of antibodies were determined in each individual case based on the diagnosed immunophenotype. Major leukemic populations and questionable monocyte-like populations were purified using a BD FACS Aria III flow sorter (BD). Sample preparation depended on the downstream molecular study. For FISH, an erythrocyte lysis buffer with fixative (FACS Lyse, BD) was used, and the presorting samples were diluted in RPMI-1640 medium (PanEco, Moscow, Russia). For clonality assessment, a nonfixative lysis agent (PharmLyse, BD) was used, and the cells were diluted in phosphate-buffered saline (Cell Wash, BD). Cells were sorted in ‘Purity’ mode and collected in Eppendorf tubes containing relevant buffer. A total of 10,000 to 15,000 cells were sorted in duplicate for FISH; 50,000 to 150,000 cells were sorted for clonality testing by multiplex PCR, and 3 to 5 million cells were sorted in duplicate for clonality by NGS.

### 4.5. Fluorescence In Situ Hybridization

As a part of standard ALL diagnostics, GTG-banded conventional karyotyping and FISH were performed on the diagnostic BM samples of all patients. BM aspirates were cultured overnight without mitogenic stimulation and were processed as described previously [33]. The most common MPAL-specific cytogenetic aberrations, t(9;22)(q34;q11)/*BCR::ABL1*, *KMT2A* and *ZNF384* gene rearrangements, were evaluated by FISH with the Kreatech ON BCR::ABL1 DCDF probe (Leica Microsystems B.V., Amsterdam, The Netherlands), Kreatech ON KMT2A::MLLT11 probe (Leica) and *ZNF384* gene break-apart probe (Cytocell, Milton, Cambridge, UK), respectively. Detected patient-specific genetic rearrangements were then analyzed in the sorted cell populations. After sorting, the cells in Eppendorf tubes were immediately centrifuged at 5000× *g* for 5 min. The supernatant was then discarded, and the cells were centrifuged for another minute under the same conditions. The remaining drop was pipetted and transferred to a microscope slide (Citotest Haimen, Jiangsu, China) and left overnight. The slides were then processed according to standard procedures.

### 4.6. Detection of IG Gene Rearrangements by Multiplex PCR

For multiplex PCR, the obtained cell suspensions were centrifuged at 5000× *g* for 5 min. The cell pellets were resuspended in proteinase K digestion buffer (50 mM KCl, Sigma-Aldrich, St. Louis, MO, USA; 100 mM Tris-HCl pH 8.3, Sigma-Aldrich; 2.5 mM MgCl_2_, Sigma-Aldrich; 0.45% Nonidet P40, Boehringer, Ingelheim am Rhein, Germany; 0.45% Tween 20; Boehringer) at a proportion of 1 μL per 500–1000 cells, frozen and stored at −20 °C. The frozen cell lysates were thawed at room temperature. After thawing, proteinase K (Macherey-Nagel, Düren, Germany) was added at a concentration of 2 units per μL, and the mixture was incubated for 3 h at 56 °C; the enzyme was inactivated by heating at 95 °C for 7 min. The mixtures were vortexed for 1 min and centrifuged at 10,000× *g* for 5 min. The obtained supernatants were used for multiplex PCR based on 2000–4000 cells per reaction. The PCR analysis of *IG* gene rearrangements was based on EuroClonality/BIOMED-2 protocols [34]. All PCRs were performed in duplicate and included polyclonal (donor-derived lymphocytes) and negative controls. Fragment analysis was performed with GeneMapper software (Thermo Fisher Scientific, Waltham, MA, USA).

### 4.7. Detection of Clonal Rearrangements of IG and TR Genes by Next-Generation Sequencing

In one case, clonality assessment was performed using high-throughput sequencing. DNA was isolated from sorted cell populations using the ExtractDNA Blood kit (Evrogen, Moscow, Russia). Sequencing libraries for detection of the clonal repertoire were prepared using two-round PCR. In the first round of PCR, five parallel multiplex PCRs were performed for each sample using a primer set for the V, D and J loci of the *IG* heavy chain (3 multiplex PCRs with primers for the FR1, FR2, FR3 segments of the *IGH* chain) and one multiplex PCR with primers for the V and J segments of the *IGK* and *IGL* light chains. For assessment of *TR* rearrangements, four multiplex PCRs were performed for each sample using a primer set for T-cell receptors (TRG, TRD, TRA and TRB loci) [35]. In each reaction, 40 ng of genomic DNA was used. In the second round of PCR, i7 and i5 indices containing adaptor sequences were added. The PCR products were purified using magnetic beads. Sequencing of the libraries was performed using an Illumina sequencer (Illumina, San Diego, CA, USA). The files obtained after sequencing and demultiplexing were analyzed using the Galaxy web platform (https://usegalaxy.org, accessed on 22 January 2021) and IgBLAST software (https://www.ncbi.nlm.nih.gov/igblast, assessed on 30 January 2021). A frequency of 5% was used as a cutoff to identify rearrangements specific for leukemic clones.

## 5. Conclusions

It can be concluded that inclusion of immunophenotype-based cell sorting with subsequent application of different molecular techniques helps in the correct diagnosis of a rare disease entity such as MPAL.

## Data Availability

The datasets generated and analyzed during the current study are available from the corresponding author on reasonable request.

## Supplementary Materials

The following supporting information can be downloaded at: https://www.mdpi.com/article/10.3390/ijms24065260/s1.
