# Supplementary material for: Recognizing Minor Leukemic Populations with Monocytic Features in Mixed-Phenotype Acute Leukemia by Flow Cell Sorting Followed by Cytogenetic and Molecular Studies: Report of Five Exemplary Cases
Source: Int J Mol Sci. 2023 Mar 9;24(6):5260. doi: 10.3390/ijms24065260 (PMC10049081; doi:10.3390/ijms24065260)
Supplement: Supplementary file 1 [file ijms-24-05260-s001.zip › ijms-2201407-supplementary.pdf]

Table S1. Patient demographics and cytological and genetic findings

Patient	Age/Sex	Morphology and Cytochemistry	Immunophenotyping		Cytogenetics
			**B-cell population**	**Monocytic/myeloid population**	
#1	5 m/F	Anaplastic blasts with lymphoid differentiation features negative for MPO, SBB, ANAE, PAS	BI, 90%: CD19+, CD79a+, CD45+, CD15+, NG2+, negative for CD10, CD22, MPO and lysozyme	7%: CD33+, CD14+, CD64+, CD45+, CD15+, weak CD19 and CD79a, negative for NG2, positive for lysozyme	46,XX, t(11;19)(q23;p13.3) [20]; positive for t(11;19) by FISH
#2	11 y/M	25%: small blasts with round nuclei, high N:CR, basophilic cytoplasm; negative for MPO, SBB, ANAE, partially positive for PAS<br>60%: intermediate to large blasts with round nuclei and variable N:CR; positive for MPO, SBB, ANAE, partially positive for PAS	BII, 42%: CD19+, CD10+, CD79a+, CD45+, CD34+, CD13+, negative for CD22, MPO and lysozyme	38%: CD33+, CD13+, CD45+, CD15+, weak CD19 and CD79a, CD34+, CD117+, positive for MPO	No metaphases; positive for t(9;22)(q34;q11) by FISH
#3	3 m/F	67-82%: small blasts with high N:CR; negative for MPO, SBB, ANAE, positive for PAS<br>3-10%: intermediate to large blasts with round nuclei and medium N:CR; negative for ANAE, positive for MPO, SBB, PAS	BI, 42%: CD19+, CD22+, CD79a+, CD45+, CD34+, CD13+, CD33+, negative for CD10, MPO and lysozyme	7%: CD33+, CD14+, CD64+, CD13+, CD45+, CD15+, weak CD19 and CD79a, positive for MPO and lysozyme	46,XX, t(12;17)(p13;q13), del(20q) [5]; positive for a *ZNF384* rearrangement by FISH

#4	18 y/M	Anaplastic blasts with lymphoid differentiation features, negative for MPO, SBB, ANAE, PAS. Poor quality of BM smears	BII, 65%: CD19+, weak CD10, CD79a+, CD45+, negative for CD22, MPO and lysozyme	7%: CD33+, CD14+, CD64+, CD13+, CD45+, weak CD19, positive for lysozyme	46,XY
#5	15 y/F	89%: small blasts with round nuclei, high N:CR, basophilic cytoplasm; negative for MPO, SBB, ANAE, partially positive for PAS 11%: intermediate blasts with round or irregular nuclei and variable N:CR, negative for MPO, ANAE, positive for SBB, partially positive for PAS	BII, 85%: CD19+, weak CD10, CD22+, CD79a+, CD45+, negative for MPO and lysozyme	12%: CD33+, CD14+, CD64+, CD13+, CD45+, weak CD19 and CD79a, positive for lysozyme	46,XX

N:CR, nucleus-to-cytoplasm ratio; MPO, myeloperoxidase; SBB, Sudan black B; PAS, periodic acid-Schiff reaction; ANAE, α-naphthyl acetate-esterase; BM, bone marrow; m, months; y, years; F, female; M, male.
